# Interferon‐Regulatory Factor 4 Is Required Not Only for Induction but Also for Maintenance of the Th17 Phenotype

**DOI:** 10.1002/eji.70193

**Published:** 2026-04-26

**Authors:** Janis Patten, Prema Erramsetti, Addi Josua Romero Olmedo, Olaf Pinkenburg, Magdalena Huber, Michael Lohoff

**Affiliations:** ^1^ Clinic for Hematology, Oncology and Immunology Philipps‐University Marburg Marburg Germany; ^2^ Molecular Physiology Philipps‐University Marburg Marburg Germany; ^3^ Institute of Systems Immunology Philipps‐University Marburg Marburg Germany

**Keywords:** cytokine, interleukin, interferon‐regulatory factor 4 (IRF4), maintenance, Th17‐cell‐differentiation

## Abstract

We previously showed that primary differentiation of naïve T cells is dependent on IRF4. Now we show that maintenance of already differentiated conventional and pathogenic Th17 cells is also dependent on IRF4.

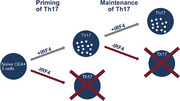

When first activated, CD4^+^ T cells differentiate into specialized T helper subtypes, dependent on the key transcription factors (TFs) T‐BET for Th1, GATA3 for Th2, PU.1 for Th9, and RORγt for Th17. In addition, our group established decisive roles for the TF interferon‐regulatory factor 4 (IRF4) in differentiation of Th2 [1], Th9 [2], and Th17 [3] cells and similar IRF4 activities in CD8+ cells. It was suspected that IRF4 fulfills these pleiotropic activities by opening chromatin for transcribing genes necessary for further differentiation [4, 5, 6].

Although these IRF4 activities on naïve T cells are well accepted, little information exists on the importance of IRF4 for maintaining the respective T‐cell phenotypes, once primary differentiation has successfully occurred. However, this information is very important in order to make IRF4 attractive for therapeutic interventions. One recent publication suggested that IRF4 is no longer necessary for maintenance [7]. However, that conclusion was based on indirect in vivo experiments and cell population data, whereas no analysis was made on the single cell level.

Herein, we set out to answer this issue more stringently in vitro for Th17 cells. The time line of the experiment is depicted in Figure 1. We used mice with one null and one floxed allele of IRF4 (IRF4fl/−). Purified CD4^+^ T cells were differentiated into conventional Th17 cells using anti‐CD3/28 and IL‐6/TGFß. Successful differentiation was confirmed after primary restimulation with PMA/ionomycin (Figure 2A). Thereafter, the majority of the restimulated cells underwent a commercial “secretion” kit that attaches secreted IL‐17 to the surface of the producing cell and stains it by PE (Figure 2B). The percentage of cells positive for PE or intracellular IL‐17 was very similar, confirming the stringency of the assay.

**FIGURE 1:**
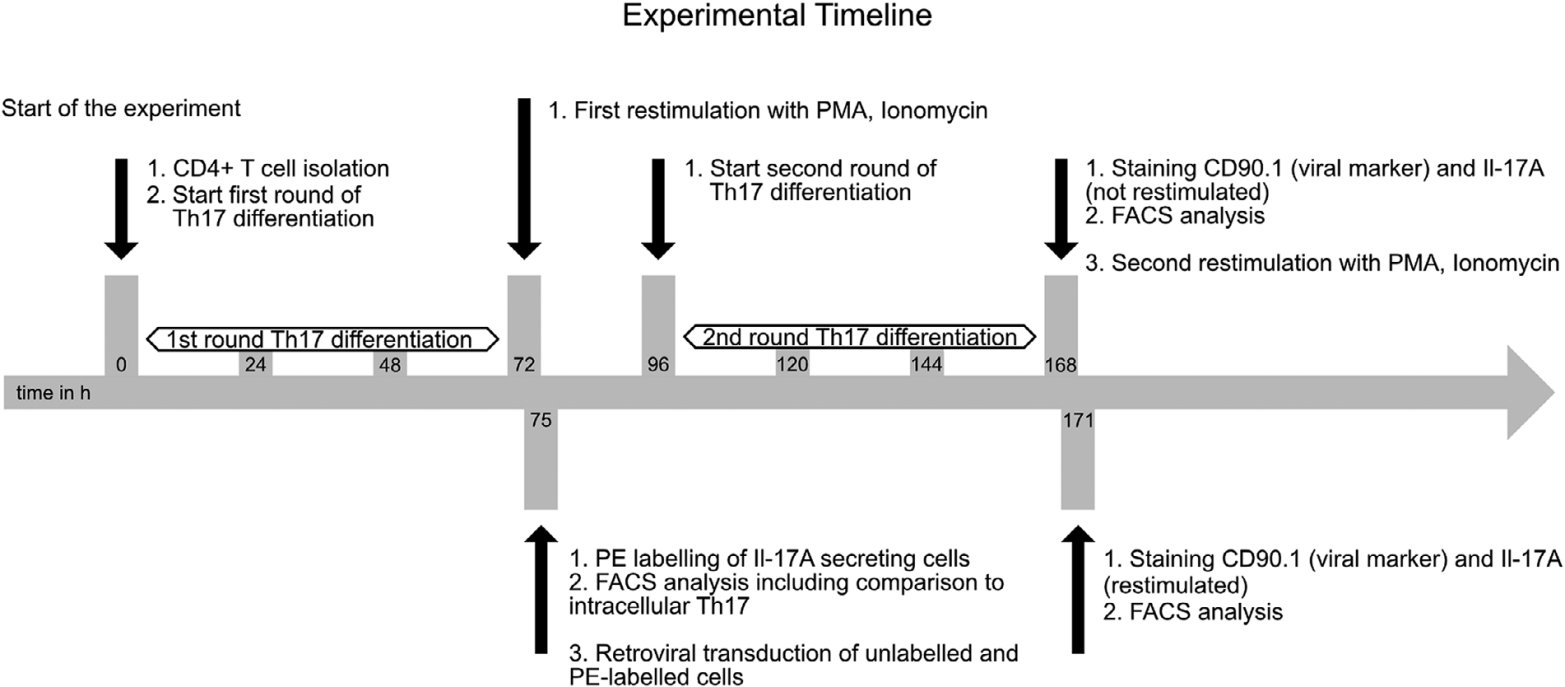
Experimental system and timeline: Purified CD4^+^ T cells from mice with one null and one floxed allele of IRF4 (IRF4fl/−) were differentiated toward Th17, then underwent a first restimulation with PMA/ionomycin with/without brefeldin A (BFA). Cells were stained for intracellular IL‐17 (+BFA) and externally (via PE; no BFA) for secreted IL‐17 and analyzed by FACS; PE‐labeled cells were then retrovirally transfected with Cre (75 h). A second round of Th17 differentiation followed (96 h). Thereafter (168 h), part of the cells were directly stained intracellularly for remaining IL‐17 production from the differentiation phase; the others underwent secondary restimulation by PMA/ionomycin plus BFA and intracellular staining for IL‐17. Ensuing FACS analysis revealed intracellular IL‐17 (APC) after secondary differentiation, successful transfection (CD90.1‐Violet), excision of the IRF4fl allele (eGFP), and IL‐17 secreted after primary differentiation (PE).

**FIGURE 2:**
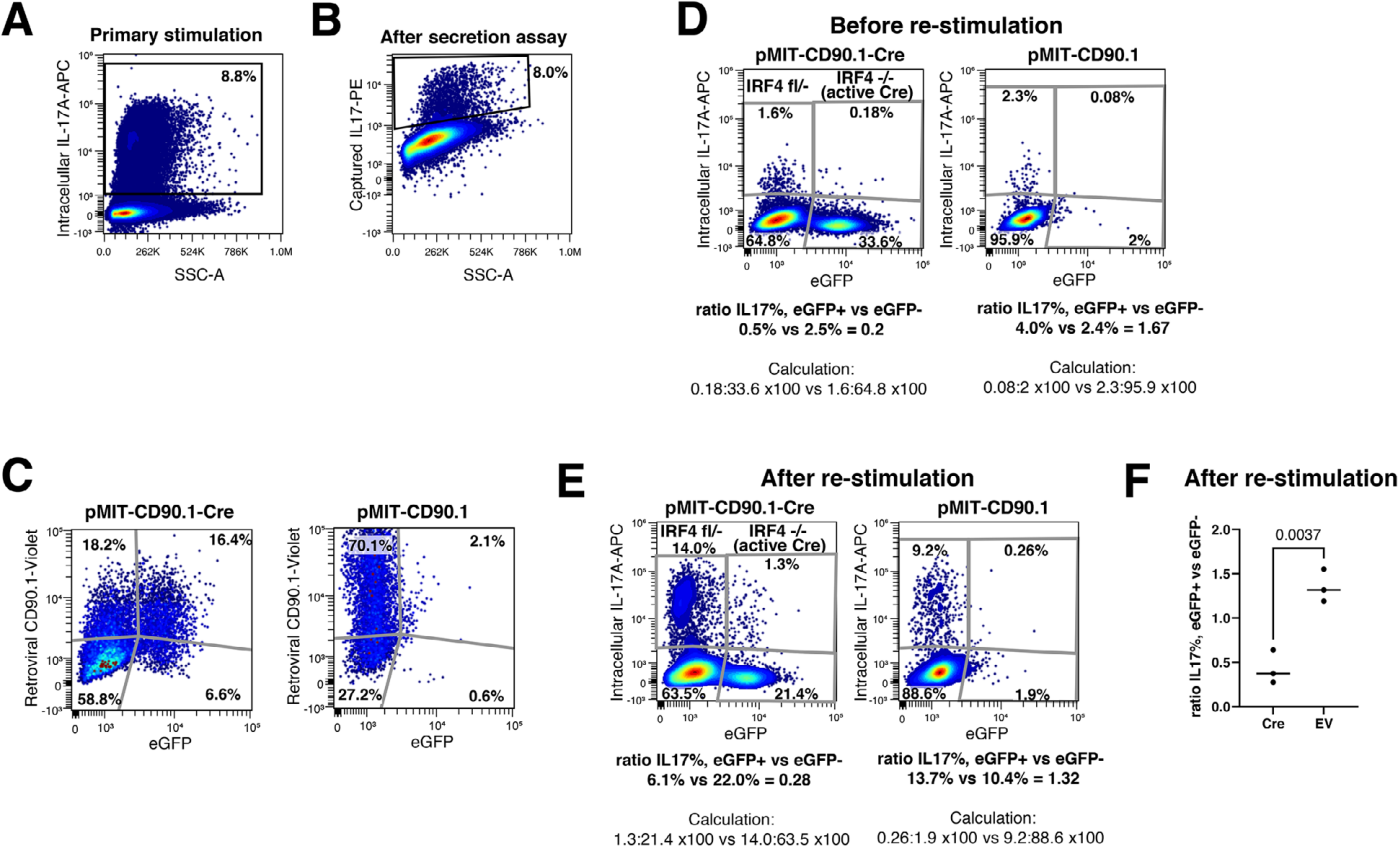
IRF4 is required for maintenance of conventional Th17 cells. (A and B) CD4^+^ T cells, purified from IRF4fl/− mice, cultured for 72 h using conventional Th17 differentiation conditions (IL‐6/TGFß) and restimulated for 3 h by PMA/ionomycin with (A) or without (B) BFA. (A) Intracellular staining for IL‐17. (B) Extracellular PE‐staining of secreted IL‐17. Numbers reflect percentages of cells positive for intracellular (A) or secreted (B) IL‐17. (C–F) Cells were replated, infected with retroviruses expressing CD90.1 plus or minus Cre, and re‐differentiated (IL‐6/TGFß) for further 72 h before staining with anti‐CD90.1 and flow cytometry. PE‐positive cells were gated. (C) *X*‐axis: cells with active Cre creating an eGFP signal from the floxed IRF4 allele; *Y*‐axis: transfected cells expressing CD90.1. (D) An aliquot of the cells described in (C) was stained for intracellular IL‐17. (E) A further cell aliquot was secondarily restimulated with PMA/ionomycin for 3 h and analyzed for intracellular IL‐17. (C–E): Numbers reflect percentages of cells in the respective quadrant; ratios of these percentages were calculated as indicated. (F) Results of the respective ratios after secondary restimulation in three separate experiments, including *p* value.

After labeling secreted IL‐17 by PE, the mixture of PE‐positive and PE‐negative cells was replated and transfected overnight with bicistronic retroviruses (RV) encoding the marker CD90.1 plus or minus Cre recombinase. Once Cre is active, the floxed IRF4 gene is removed leading (along with the null allele) to a full IRF4 knockout and eGFP staining. After transfection, the cells were further incubated for 20 h to allow for Cre to operate, before a second round of Th17 differentiation was initiated. As shown in Figure 2C, successful transfection was easily mirrored by staining for CD90.1 that was comparably detectable after this second round in cells transfected with Cre encoding or empty control RV. As expected, no significant eGFP signal was noted in cells transfected with empty control vector. A final step involved intracellular staining of IL‐17 before and after secondary restimulation by PMA/ionomycin. Altogether, the cells could now be analyzed for successful transfection (via CD90.1), active Cre (via eGFP), current IL‐17 production, and previous Th17 history via a remaining PE signal from the IL‐17 secretion assay. The overall gating strategy is depicted in Figure S1, which also confirms stability of the PE signal after the second round of Th17 differentiation. Data in Figure S1 also confirm that the capacity to produce intracellular IL‐17 after secondary restimulation was still strongly correlated with remaining PE‐staining and thus previous IL‐17 production (see histograms), excluding any artifacts such as hypothetical diffusion of PE from one cell to another.

We then compared PE‐labeled cells with versus without IRF4 for their capacity to further produce IL‐17 (Figure 2D,E). To do this, we calculated separate percentages of IL‐17 positivity per eGFP+ (e.g., 1.3/21.4 = 6.1%) or eGFPneg cells (e.g., 14.0/63.5 = 22%). In control cells without Cre, we created a similar calculation for cells with or without a dim, likely autofluorescent eGFP signal. It became obvious that whenever Cre was active, that is, the remaining IRF4 allele was removed and cells became eGFP positive, the capacity of cells to produce IL‐17 sharply dropped. This was already visible before secondary restimulation (Figure 2D, IL‐17% in IRF4fl/− 2.5% vs. IL‐17% in IRF4−/− 0.5%), illustrating remaining IL‐17 production in few cells from the second differentiation period. Secondary restimulation by PMA/ionomycin strongly induced de novo IL‐17 production. Importantly, all cells analyzed in this experimental part were gated for PE‐positivity and thus entirely reflected previously IL‐17‐producing cells, which mostly had, meanwhile, stopped IL‐17 production without secondary restimulation. Thus, their de novo IL‐17 production correctly reflected the maintenance of the Th17 phenotype, which decisively depended on further IRF4 activity (Figure 2E, IL‐17% in IRF4fl/− 22.0% vs. IL‐17% in IRF4−/− 6.1%). In control cells with no Cre, the respective percentages were 2.4% versus 4.0% (Figure 2D) and 10.4% versus 13.7% (Figure 2E), reflecting remaining IRF4 activity. Cre was not toxic, because in PE+CD90.1+eGFPneg cells, IL‐17 production after secondary restimulation was similar as in PE+CD90.1neg uninfected cells (Figure S2). Due to the bicistronic nature of the retrovirus that encodes CD90.1 only after successful transcription of Cre, CD90.1+eGFPneg cells express Cre but lack Cre activity. Of note, we used on purpose a Cre version lacking a nuclear‐localization signal, exactly to allow for appearance of such CD90.1+eGFPneg cells as control.

Importantly, these results were reproduced in three independent experiments with high statistical significance. To prove this, we formed ratios of the mentioned percentages of IL‐17+ cells, as indicated in Figure 2D,E and merged these ratios for the three experiments (Figure 2F).

We repeated this experiment with so‐called pathogenic Th17 cells raised with IL‐1β/IL‐6/IL‐23 instead of IL‐6/TGFß. Thus, IRF4 is also mandatory for primary (previously not shown) and persistant differentiation of pathogenic Th17 cells (Figure S3).

Together, our data point to a continuous necessity of IRF4 activity for maintenance and not just initiation of Th17 cell differentiation. We consider this information as highly relevant, because IRF4 can be considered a potential target in diseases with pathogenic activity of Th17 cells, like in colitis or arthritis. As most patients develop disease only once the pathogenic cells have already differentiated, a role of IRF4 for maintenance of the cell phenotype is a pre‐requirement for any therapeutic considerations involving it.

## Conflicts of Interest

The authors declare no conflicts of interest.

## Supporting information




**Supporting File**: eji70193‐sup‐0001‐SuppMat.docx.

## Data Availability

The study data are available from the corresponding author upon request.
