# Supplementary material for: Interferon‐Regulatory Factor 4 Is Required Not Only for Induction but Also for Maintenance of the Th17 Phenotype
Source: Eur J Immunol. 2026 Apr 26;56:e70193. doi: 10.1002/eji.70193 (PMC13111725; doi:10.1002/eji.70193)
Supplement: Supplementary file 1 — Supporting File: eji70193‐sup‐0001‐SuppMat.docx. [file EJI-56-e70193-s001.docx]

# SUPPLEMENTARY MATERIAL

**Interferon-regulatory factor 4 is required not only for induction but also for maintenance of the Th17 phenotype**

Janis Patten^1*^, Prema Erramsetti^1*^, Addi Josua Romero Olmedo ^1^, Olaf Pinkenburg^2^, Magdalena Huber^3^, Michael Lohoff^1^

^1^Clinic for Hematology, Oncology and Immunology, ^2^Molecular Physiology and ^3^Institute of Systems Immunology, Philipps-University Marburg, Marburg, Germany

**Corresponding Author:** Michael Lohoff (lohoff@med.uni-marburg.de)

***: equal contribution**

**Methodology:**

- 1. Mice
     IRF4fl/- mice were bred in our facilities by mating IRF4fl/fl [1] with homozygous IRF4 knockout mice  [2]. Spleen and lymph nodes of female mice were harvested at the age of 12-16 weeks. Organs were then crushed, sieved and CD4+ T cells were prepared using the mouse CD4+ T Cell Isolation Kit (Miltenyi Biotec).
  2. Viral supernatant generation
     The  pMIT-CD90.1-Cre plasmid was constructed based on pMIG-Cre [3], kindly provided by Hassan Jumaa (University of Ulm). We removed the second NcoI cutting side in pMIG-Cre via site-directed  mutagenesis as described in [4] using the forward primer 5’- cga ggt tgt ggt taa tta agg atg gcc aat tta ctg acc gt - 3’  and reverse primer 5’- acg gtc agt aaa ttg gcc atc ctt aat taa cca caa  cct cg - 3’(biomers).
     The generated plasmid was then opened with the enzymes NcoI and  SalI (Thermo Fisher). A Thy1.1. (CD90.1) sequence was generated via PCR  from pMIT-CD90.1 [5] using the forward primer 5’- ttt ggt ctc cca tga acc cag cca tca gcg  tcg c -3’ and reverse primer 5’-  ttt tgt cga ctc aca gag aaa tga agt  cca ggg ctt gga gg - 3’ (biomers) which was then digested with BsaI  and SalI (Thermo Fisher) and ligated into the opened plasmid.
     For the generation of viral particles from pMIT-CD90.1-Cre and pMIT-CD90.1, the constructs used were transfected together with pEco and pCGP into HEK293T cells using the calcium phosphate transfection method as previously described [4].  8h after transfection, the cells were washed with BSS and fresh RPMI medium was added. After 24h and 48h, the supernatant was harvested, aliquoted and frozen at -80°C.

1.3 Th17 differentiation
Purified CD4+ T cells were seeded in anti-CD3 coated [6] 48-well cell culture plates using IMDM (Sigma–Aldrich) medium supplemented with 10% FCS (Sigma),  1% Penicillin/Streptomycin (GIBCO), 1% L-Glutamine (Sigma), and 55mM  β-mercaptoethanol (GIBCO). For Th17 differentiation 2 μg/ml anti-CD28, 5 μg/ml αIFNy and 10% αIL-4 solution, 2 ng/ml rhTGFß1 and 50 ng/ml rmIL-6  were added and cells were cultured for 72h.

For pathogenic Th17 differentiation, 2µg/mL anti-CD28, 5µg/ml α IFNγ and 10% αIL-4 solution, 50ng/ml rmIL-6, 20ng/ml rmIL-23 and 25ng/ml rmIL-1β were added and cells also were cultured for 72hrs.

1.4 PE labelling of IL-17 secreting cells
Differentiated cells were harvested, washed in PBS and restimulated (first restimulation as opposed to second restimulation described in 1.5) using 50 ng/ml PMA, 750 ng/ml ionomycin (all from Sigma-Aldrich) in IMDM for 3h. IL-17 secreting cells were then labelled externally with PE using the Mouse IL-17 Secretion  Assay – Detection Kit (PE) (Miltenyi Biotec) according to the manufacturer’s instructions.
To control for differentiation, a portion of cells was restimulated using 50 ng/ml PMA, 750 ng/ml ionomycin, however also using 5 μg/ml brefeldin A (all  from Sigma-Aldrich) in IMDM for 3h and fixated with 2% PFA (Alfa Aesar)  for 10 minutes; cells were permeabilized with saponin buffer and intracellularly stained for 15 min using αIL17A (eBio17B7, Invitrogen) diluted 1:500 in saponin buffer. The rate of PE positively labelled, and therefore IL-17 secreting T cells was then checked using flow cytometry (see below) and compared with the rate of intracellularly stained IL-17A+ T cells.

1.5. Spin transduction and further culture
For spin transduction, PE-labelled cells obtained after the secretion assay were then centrifuged for 1.5h at 1500G and 37°C [7] in cell culture supernatants containing viral particles of pMIT-CD90.1 or pMIT-CD90.1-Cre together with 50 U/ml recombinant human (rh) IL2 (Peprotech, Hamburg, Germany) and 10 μg/ml polybrene (Sigma–Aldrich, St. Louis,  USA) followed by an overnight culture in IMDM (Sigma–Aldrich) supplemented as described above. Thereafter, PE-labelled and transfected cells were again cultured for 72h in IMDM medium supplemented with the same Th17 cell differentiation reagents as described above for the initial Th17 differentiation.

After this second culture period, cells were harvested and washed in PBS. A portion of cells was fixated without further restimulation and stained for intracellular IL17A as described above to check for remaining IL17A production from the previous culture period. Another portion of cells was submitted to a second step of restimulation, again using 50 ng/ml PMA, 750 ng/ml ionomycin and 5 μg/ml brefeldin A (all from Sigma-Aldrich) in IMDM for 3h. Thereafter, cells were surface-stained in PBS/1% FCS for CD90.1 using Brilliant Violet 421-conjugated anti-rat CD90/mouse CD90.1 (Thy-1.1) Antibody (Biolegend) to check for the transfection rate, then fixated with 2% PFA (Alfa Aesar) for 10 minutes and intracellularly stained for IL17A.

1.6. Flow cytometry analysis

Flow cytometry data was acquired using an AttuneTM NxT Flow Cytometer (Thermo Fisher Scientific Inc., Waltham, USA). Flow cytometry experiments followed the “Guidelines for the use of flow cytometry and cell sorting in immunological studies”[8].

1.7 Statistical analysis
Statistical analysis was performed using the GraphPad PRISM (version 9). This involved the application of an unpaired, two-tailed Student‘s t-test. A p-value of less than 0.05 was considered to be statistically significant.

**Author contribution:**

JP and PE performed all experiments, AJRO helped with flow cytometric analyses, OP helped with cloning, MH helped with retroviral supernatant generation and ML designed experiments and prepared the publication.


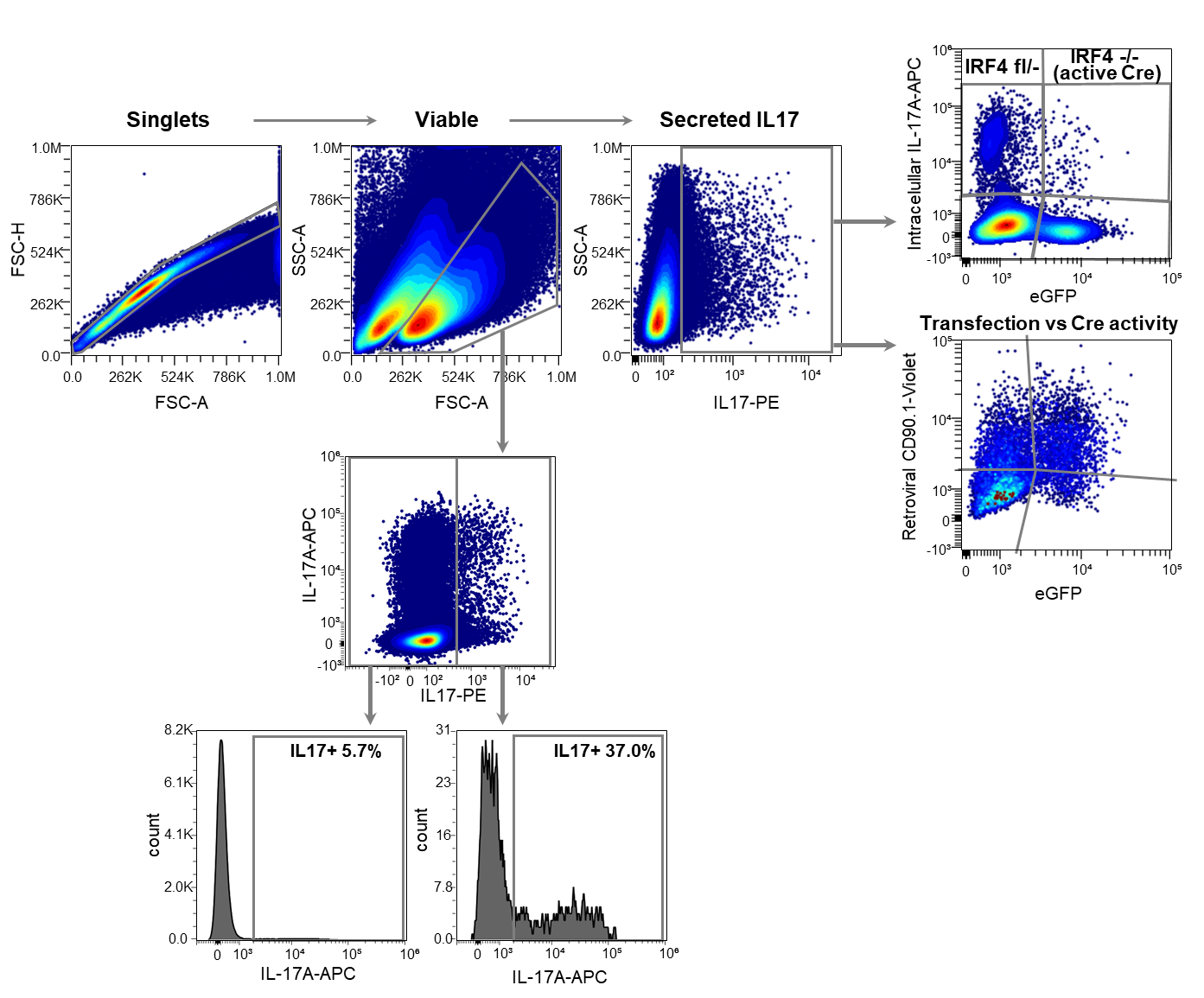


**Supplementary Figure 1**: Gating strategy for the cell analysis described in Fig.2, performed after 3h of secondary restimulation by PMA/ionomycin (171 h in Figure 1). IL17 PE characterizes cells still stably stained for secreted IL17 3 days after performing the secretion assay (gate setting for PE according to control cells not stained by PE in the secretion assay). In one panel, de novo intracellular IL17 production is plotted against previous secretion of IL17 after primary stimulation (reflected by still ongoing PE staining). The histograms confirm the correlation of preferential de novo IL17 production capacity with PE staining (right histogram) compared to cells not stained by PE (left histogram).

**
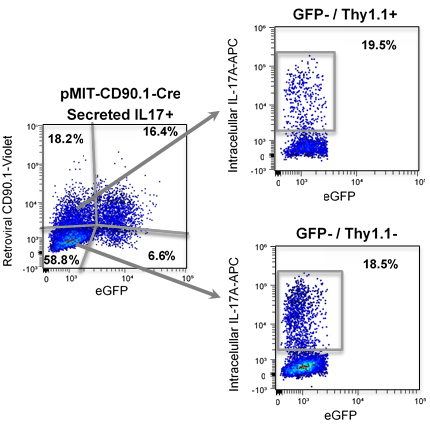
**

**Supplementary Figure 2: Lack of Cre toxicity.** PE+ cells depicted in sFig.1 (“secreted IL17”) and gated for CD90.1 and eGFP staining, were subgated to separately analyse IL17 production in GFP- cells, which were or were not transduced by Cre. In CD90.1+ eGFP- cells, Cre is present but inactive.

**
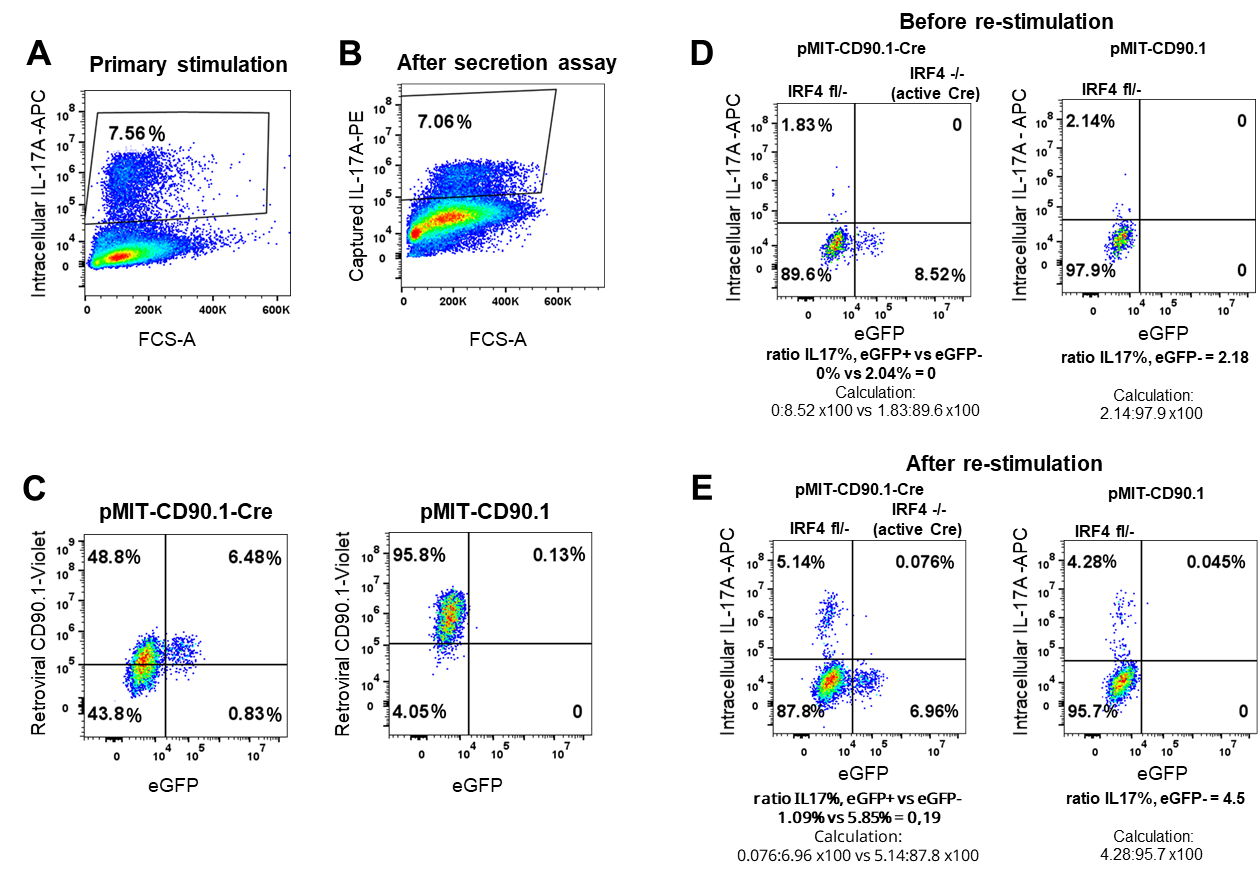
Supplementary Figure 3: IRF4 is required for maintenance of pathogenic Th17 cellls.** An experiment similar to the one described in Fig.2 was performed, however using pathogenic instead of conventional Th17 differentiation conditions. (A,B) CD4+ T cells, purified from IRF4 fl/- mice, cultured for 72h in the presence of IL1β/IL6/IL23 and restimulated for 3h by PMA/Ionomycin with (A) or without (B) BFA. (A) Intracellular staining for IL-17. (B) Extracellular PE-staining of secreted IL-17. Numbers reflect percentages of positive cells. (C-E) Cells were replated, infected with retroviruses expressing CD90.1 plus or minus Cre and re-differentiated ((IL1β/IL6/IL23)) for further 72h before staining with anti-CD90.1 and flow cytometry. PE positive cells were gated. (C) X-axis: cells with active Cre creating an eGFP signal; Y-axis: Transfected cells expressing CD90.1. (D) An aliquot of the cells described in (C) was stained for intracellular IL-17. (E) A further cell aliquot was secondarily restimulated with PMA/Ionomycin for 3h and then analysed for intracellular IL-17. (C-E): Numbers reflect percentages of cells in the respective quadrant; ratios of these percentages were calculated as indicated. Two independent experiments with similar outcome. In the second experiment, the numbers with Cre virus were: eGFP- IL17+:7.3%, eGFP- IL17-: 87.6%, eGFP+ IL17+: 0% and eGFP+ IL17-: 5.2%. In the empty vector control, the percentage of eGFP+ cells was zero, of eGFP-IL17+: 5.6%. of eGFP-IL17-: 94.4%.

**References:**

1 **Klein, U., Casola, S., Cattoretti, G., Shen, Q., Lia, M., Mo, T., Ludwig, T., Rajewsky, K. and Dalla-Favera, R.,** Transcription factor IRF4 controls plasma cell differentiation and class-switch recombination. *Nat.Immunol.* 2006. **7**: 773-782.

2 **Mittrucker, H. W., Matsuyama, T., Grossman, A., Kundig, T. M., Potter, J., Shahinian, A., Wakeham, A., Patterson, B., Ohashi, P. S. and Mak, T. W.,** Requirement for the transcription factor LSIRF/IRF4 for mature B and T lymphocyte function. *Science* 1997. **275**: 540-543.

3 **Reuther, P., Gopfert, K., Dudek, A. H., Heiner, M., Herold, S. and Schwemmle, M.,** Generation of a variety of stable Influenza A reporter viruses by genetic engineering of the NS gene segment. *Sci Rep* 2015. **5**: 11346.

4 **Kang, C. H., Hartmann, E., Menke, L., Staudenraus, D., Abass, E. F., Raifer, H., Porapu, A., Camara, B., Brustle, A., Pinkenburg, O., Bieringer, M. and Lohoff, M.,** A hyperactive mutant of interferon-regulatory factor 4. *Eur J Immunol* 2019. **49**: 812-815.

5 **Bothur, E., Raifer, H., Haftmann, C., Stittrich, A. B., Brustle, A., Brenner, D., Bollig, N., Bieringer, M., Kang, C. H., Reinhard, K., Camara, B., Huber, M., Visekruna, A., Steinhoff, U., Repenning, A., Bauer, U. M., Sexl, V., Radbruch, A., Sparwasser, T., Mashreghi, M. F., Wah Mak, T. and Lohoff, M.,** Antigen receptor-mediated depletion of FOXP3 in induced regulatory T-lymphocytes via PTPN2 and FOXO1. *Nat Commun* 2015. **6**: 8576.

6 **Lohoff, M., Dirks, M., Rohwer, P. and Rollinghoff, M.,** Studies on the mechanism of polyclonal B cell stimulation by TH2 cells. *Eur J Immunol* 1989. **19**: 77-81.

7 **Staudenraus, D., Porapu, A., Leister, H., Gupta, D. D. and Lohoff, M.,** Point mutation L116R in interferon-regulatory factor 4 differentially impacts key cytokine production in Th2, Th9, and Th17 cells. *European Journal of Immunology* 2022. **52**: 1680-1683.

8 **Cossarizza, A., Chang, H. D., Radbruch, A., Acs, A., Adam, D., Adam-Klages, S., Agace, W. W., Aghaeepour, N., Akdis, M., Allez, M., Almeida, L. N., Alvisi, G., Anderson, G., Andra, I., Annunziato, F., Anselmo, A., Bacher, P., Baldari, C. T., Bari, S., Barnaba, V., Barros-Martins, J., Battistini, L., Bauer, W., Baumgart, S., Baumgarth, N., Baumjohann, D., Baying, B., Bebawy, M., Becher, B., Beisker, W., Benes, V., Beyaert, R., Blanco, A., Boardman, D. A., Bogdan, C., Borger, J. G., Borsellino, G., Boulais, P. E., Bradford, J. A., Brenner, D., Brinkman, R. R., Brooks, A. E. S., Busch, D. H., Buscher, M., Bushnell, T. P., Calzetti, F., Cameron, G., Cammarata, I., Cao, X., Cardell, S. L., Casola, S., Cassatella, M. A., Cavani, A., Celada, A., Chatenoud, L., Chattopadhyay, P. K., Chow, S., Christakou, E., Cicin-Sain, L., Clerici, M., Colombo, F. S., Cook, L., Cooke, A., Cooper, A. M., Corbett, A. J., Cosma, A., Cosmi, L., Coulie, P. G., Cumano, A., Cvetkovic, L., Dang, V. D., Dang-Heine, C., Davey, M. S., Davies, D., De Biasi, S., Del Zotto, G., Dela Cruz, G. V., Delacher, M., Della Bella, S., Dellabona, P., Deniz, G., Dessing, M., Di Santo, J. P., Diefenbach, A., Dieli, F., Dolf, A., Dorner, T., Dress, R. J., Dudziak, D., Dustin, M., Dutertre, C. A., Ebner, F., Eckle, S. B. G., Edinger, M., Eede, P., Ehrhardt, G. R. A., Eich, M., Engel, P., Engelhardt, B., Erdei, A.,** Guidelines for the use of flow cytometry and cell sorting in immunological studies (second edition). *Eur J Immunol* 2019. **49**: 1457-1973.
